# Racial, Sex, and Age Disparities in Cardiomyopathy Etiology: A Social Determinant Analysis of 366 Cardiac Patients

**DOI:** 10.7759/cureus.95664

**Published:** 2025-10-29

**Authors:** Kiki J Estes-Schmalzl, Amy J Marcano-Reik, Kristin Lefebvre

**Affiliations:** 1 Nursing, Messiah University, Mechanicsburg, USA; 2 Clinical Research, University of Jamestown, Fargo, USA; 3 Clinical Research and Health Sciences, University of Jamestown, Fargo, USA

**Keywords:** dilated cardiomyopathy, gender disparities, health equity, heart failure, hypertrophic cardiomyopathy, hypertrophic cardiomyopathy (hcm), peripartum cardiomyopathy, racial and ethnic disparities, social determinants of health (sdoh)

## Abstract

Background: Inequities in heart failure persist across race, sex, and age, but etiology-specific patterns in cardiomyopathies remain underexplored. We examined disparities across cardiomyopathy etiologies using a social determinants of health (SDoH) lens.

Methods: We analyzed 366 left ventricular tissue samples from the GEO dataset GSE141910, focusing on cardiomyopathy subtypes: dilated cardiomyopathy (DCM), hypertrophic cardiomyopathy (HCM), peripartum cardiomyopathy (PPCM), and nonfailing controls. Demographics included race (African American, Caucasian), sex, and age. We compared distributions using χ² or Fisher's exact tests and assessed age differences with t-tests/analysis of variance.

Results: African Americans showed a 1.69-fold higher prevalence of DCM vs. Caucasians (62.1%, n = 77 vs. 36.8%, n = 89; χ² = 27.101, df = 2, p < 0.001, Cramér's V = 0.272) and presented 5.9 years earlier (48.9 ± 12.9 years, n = 77 vs. 54.8 ± 7.1 years, n = 89; p < 0.001, Cohen's d = -0.572). HCM was 13.8-fold more common in Caucasians (11.2%, n = 27 vs. 0.8%, n = 1; Fisher's exact p < 0.001). PPCM occurred exclusively in women and was nearly twofold more frequent among African Americans (62.5%, n = 5 of 8 PPCM cases), who also presented younger than Caucasians (29.5 years, n = 2 vs. 37.2 years, n = 4). Male sex was associated with higher DCM prevalence (51.5%, n = 100 vs. 38.4%, n = 66; χ² = 13.845, df = 3, p = 0.016, Cramér's V = 0.194) and predominated in HCM (60.7%, n = 17). Intersectional analyses revealed disproportionate burden among younger African Americans; among DCM patients <45 years, 71.4% (n = 15 of 21) were African Americans.

Conclusions: Cardiomyopathy etiology is associated with systematic demographic patterns. African Americans are more likely to experience earlier onset and greater DCM burden, while Caucasians exhibit markedly higher HCM prevalence. Male sex is disproportionately represented in DCM and HCM. These findings are consistent with SDoH-driven inequities and underscore the need for earlier detection, equity-centered prevention, and improved access to specialized diagnostics and therapies.

## Introduction

Heart failure (HF) affects an estimated 6.7 million Americans and is projected to increase substantially in the coming decades, with concomitant growth in deaths and disability [1,2]. The burden is unequally distributed: younger populations and racial and ethnic minority groups experience higher incidence, earlier onset, greater hospitalization rates, and worse outcomes [1,3-5]. Social determinants of health (SDoH), including structural racism, socioeconomic disadvantage, and barriers to care, shape exposure, risk, access to guideline-directed therapies, and ultimately survival [4,6-9]. Recent work further documents excess HF mortality and years of potential life lost among Black Americans compared with White Americans [10] and widening rural-urban inequities [1,9,11,12].

Despite this evidence, etiology-specific demographic patterns in cardiomyopathies remain underexplored. This study focuses specifically on cardiomyopathy subtypes (dilated cardiomyopathy (DCM), hypertrophic cardiomyopathy (HCM), and peripartum cardiomyopathy (PPCM)) rather than HF from all causes (such as coronary artery disease or hypertensive heart disease). Focusing only on overall HF can mask meaningful differences across these specific cardiomyopathy etiologies. Intersectionality across race, sex, and age may compound vulnerabilities, particularly among younger Black adults and Black women during reproductive years [9,13-15].

Study objectives

This study aims to quantify demographic disparities across cardiomyopathy etiologies in a diverse cohort, specifically examining distributions by race and sex, and age at presentation within etiologies and interpret findings within an SDoH framework to inform equity-centered prevention and care for these specific cardiomyopathy subtypes.

## Materials and methods

Study design and data source

This cross-sectional retrospective analysis used publicly available, deidentified data from the GEO dataset GSE141910 (National Center for Biotechnology Information, Bethesda, Maryland, United States), comprising left ventricular myocardial tissue samples collected from multiple centers. The dataset includes samples from patients who underwent cardiac transplantation, left ventricular assist device (LVAD) placement, or cardiac biopsy, as well as tissue from nonfailing donor hearts.

Study population and sampling

Inclusion Criteria

Inclusion criteria encompassed all patients in the dataset with documented cardiomyopathy diagnoses, including DCM, HCM, and PPCM, as well as nonfailing donor controls. All cases had available demographic data, including race, sex, and age at tissue collection.

Control Group

The control group consisted of nonfailing donor hearts from individuals who were approved as organ donors but whose hearts were deemed unsuitable for transplantation for noncardiac reasons (e.g., size mismatch with recipient, blood type incompatibility, logistical constraints, or donor age considerations) but without evidence of cardiac pathology on histologic examination. All control samples underwent pathological review to confirm the absence of structural heart disease, cardiomyopathy, or significant coronary artery disease.

Exclusion Criteria

Cases with incomplete demographic data or indeterminate etiology were excluded from analysis. The dataset did not include other HF etiologies such as ischemic cardiomyopathy, hypertensive heart disease, or valvular heart disease. This study, therefore, focuses exclusively on cardiomyopathy subtypes.

Sample size considerations

The study analyzed all available cases in the dataset (n = 366). Post hoc power analysis indicated adequate power (>80%) to detect the observed effect sizes for the primary comparisons (DCM by race, HCM by race), though limited power for subgroup analyses such as PPCM and African American HCM cases due to small cell sizes (n < 10). These limitations are acknowledged in interpretation.

Variables and definitions

Demographics included race (African American and Caucasian, categories as coded in the original dataset metadata), sex (female, male), and age at tissue collection. HF etiologies were classified based on clinical diagnoses provided in dataset metadata, which included detailed phenotyping from contributing centers' clinical records and imaging studies.

Statistical analysis

Primary outcomes were 1) etiology-specific distributions by race and sex, and 2) age at presentation within etiologies by race. Categorical variables were summarized as counts and percentages; continuous variables were summarized as means and standard deviations, or medians and interquartile ranges, as appropriate.

Group differences were assessed using χ² tests for categorical variables (or Fisher's exact test when expected cell counts were <5) and independent t-tests or one-way analysis of variance with Tukey's post hoc comparisons for continuous variables. Normality was assessed using Shapiro-Wilk tests and visual inspection of Q-Q plots. Effect sizes included prevalence ratios, percentage-point differences, odds ratios for sparse cells, and Cohen's d for age differences. Statistical significance was set at α = 0.05 (two-tailed).

All analyses were planned a priori and aligned with contemporary SDoH frameworks [6-8]. Analyses were conducted using R statistical software (version 4.3.0; R Foundation for Statistical Computing, Vienna, Austria) with packages including base R, dplyr, ggplot2, and GEOquery.

Ethical considerations

Because the dataset is deidentified and publicly available, the analysis is exempt from institutional review board oversight under federal guidelines (45 CFR 46.104(d)(4)) for secondary analysis of public data.

## Results

The cohort included 366 participants: 33.9% African American (n = 124) and 66.1% Caucasian (n = 242); and 53.0% male participants (n = 194) and 47.0% female participants (n = 172). Mean age was 53.3 ± 14.5 years (range 15-83). Etiologies were DCM (45.4%; n = 166), HCM (7.7%; n = 28), PPCM (1.6%; n = 6), and controls (45.4%; n = 166).

Racial disparities

Etiology distributions differed significantly by race (χ² = 27.10, df = 2, p < 0.001, Cramér's V = 0.272). African Americans exhibited a 1.69-fold higher prevalence of DCM than Caucasians (62.1%, n = 77 vs. 36.8%, n = 89; 25.3-point difference). Conversely, HCM was 13.8-fold more common among Caucasians than African Americans (11.2%, n = 27 vs. 0.8%, n = 1; Fisher's exact p < 0.001, odds ratio = 0.07, 95% confidence intervals, CI: 0.00-0.40). Among controls, Caucasians predominated (73.5%, n = 122) relative to African Americans (26.5%, n = 44).

Age disparities

African Americans with DCM presented 5.9 years earlier on average than Caucasians (48.9 ± 12.9 years, n = 77 vs. 54.8 ± 7.1 years, n = 89; t = -3.532, df = 164, p < 0.001; Cohen's d = -0.572, 95% CI: -9.1 to -2.6 years). Among controls, African Americans were also younger (50.0 ± 15.8 years, n = 44 vs. 58.1 ± 12.6 years, n = 122; t = -3.046, df = 164, p = 0.003; d = -0.595, 95% CI: -13.3 to -2.8 years). In PPCM, African American women were younger than Caucasian women by 7.8 years (29.5 ± 8.5 years, n = 2 vs. 37.2 ± 9.1 years, n = 4; sample size too small for statistical testing).

Sex disparities

**Figure 1:**
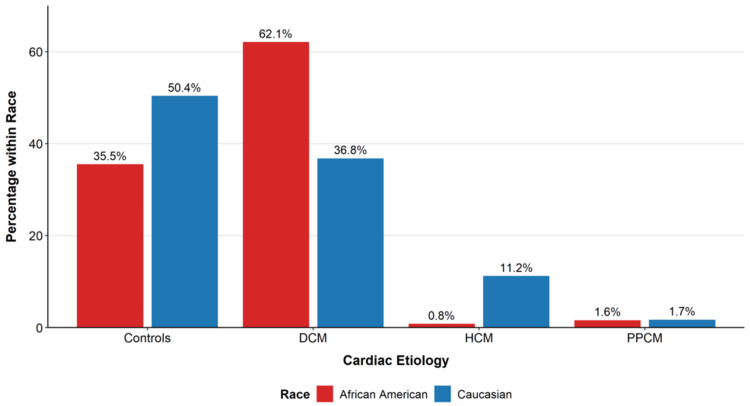
Distribution of cardiac etiologies by race (n = 366), showing the percentage of each racial group with each condition African American participants (red bars) demonstrate a higher prevalence of dilated cardiomyopathy (DCM; 62.1% vs. 36.8%) while Caucasian participants (blue bars) show a higher prevalence of hypertrophic cardiomyopathy (HCM; 11.2% vs. 0.8%). PPCM and control distributions are also shown. Overall racial differences across etiologies were statistically significant (χ² = 27.10, df = 2, p < 0.001, Cramér's V = 0.272) p < 0.001 for racial differences across etiologies DCM: dilated cardiomyopathy; HCM: hypertrophic cardiomyopathy; PPCM: peripartum cardiomyopathy

**Figure 2:**
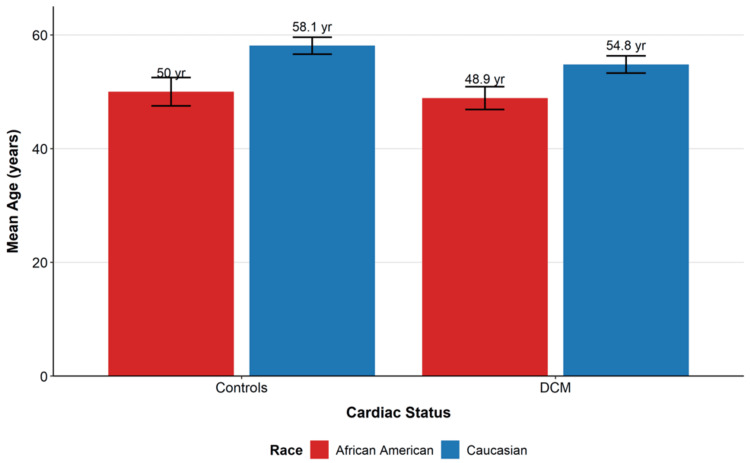
Mean age at presentation by race and etiology Mean age at presentation for African American (red bars) and Caucasian (blue bars) participants across cardiac etiologies. African Americans with DCM presented 5.9 years earlier than Caucasians (48.9 ± 12.9 vs. 54.8 ± 7.1 years; t = -3.532, df = 164, p < 0.001, Cohen's d = -0.572). Similarly, among controls, African Americans were younger than Caucasians (50.0 ± 15.8 vs. 58.1 ± 12.6 years; t = -3.046, df = 164, p = 0.003, Cohen's d = -0.595). Error bars represent standard error; p < 0.001 for both comparisons DCM: dilated cardiomyopathy

Overall sex-etiology associations were significant (χ² = 13.845, df = 3, p = 0.003, Cramér's V = 0.194). DCM was more prevalent among male participants than female participants (51.5%, n = 100 vs. 38.4%, n = 66; p = 0.016), and HCM showed male predominance (60.7%, n = 17 vs. 39.3%, n = 11). Controls were more often female participants (53.6%, n = 89 vs. 46.4%, n = 77). Intersectional patterns were marked: among DCM cases <45 years (n = 21), 71.4% (n = 15) were African American, despite representing 33.9% of the overall cohort. Table 1 summarizes sample characteristics by etiology, and Table 2 presents age by race within etiologies. Figure 1 visualizes the distribution of etiology by race, and Figure 2 shows the mean age at presentation by race and etiology.

**Table 1:** Demographic characteristics by cardiac etiology Age at presentation refers to age at time of tissue collection (cardiac transplantation, LVAD placement, or biopsy for cardiomyopathy cases; age at time of organ donation for controls) DCM: dilated cardiomyopathy; HCM: hypertrophic cardiomyopathy; PPCM: peripartum cardiomyopathy; LVAD: left ventricular assist device; SD: standard deviation

Characteristic	DCM (n = 166)	HCM (n = 28)	Controls (n = 166)	PPCM (n = 6)
Age at presentation, mean ± SD	52.1 ± 10.6	48.7 ± 12.6	55.9 ± 14.0	34.7 ± 10.3
Race, n (%)				
African American	77 (46.4)	1 (3.6)	44 (26.5)	2 (33.3)
Caucasian	89 (53.6)	27 (96.4)	122 (73.5)	4 (66.7)
Sex, n (%)				
Female	66 (39.8)	11 (39.3)	89 (53.6)	6 (100.0)
Male	100 (60.2)	17 (60.7)	77 (46.4)	0 (0.0)

**Table 2:** Age at presentation by race and cardiac etiology Age at presentation refers to age at the time of tissue collection ^*^Sample size too small for statistical inference DCM: dilated cardiomyopathy; HCM: hypertrophic cardiomyopathy; PPCM: peripartum cardiomyopathy; SD: standard deviation

Etiology	African American, mean ± SD (n)	Caucasian, mean ± SD (n)	p value	Cohen’s d
DCM	48.9 ± 12.9 (77)	54.8 ± 7.1 (89)	<0.001	-0.572
HCM	- (1)	48.7 ± 12.6 (27)	-	-
Controls	50.0 ± 15.8 (44)	58.1 ± 12.6 (122)	0.003	-0.595
PPCM^*^	29.5 ± 8.5 (2)	37.2 ± 9.1 (4)	-	-

## Discussion

Main findings

This multicenter analysis demonstrates systematic, etiology-specific demographic patterns in cardiomyopathies that are consistent with SDoH-mediated risk and access pathways. African Americans were associated with a substantially higher DCM burden and presented nearly six years earlier than Caucasians, with overrepresentation among younger patients, patterns echoing surveillance data showing earlier onset, higher hospitalizations, and rising mortality among Black adults [1,3-5]. The PPCM gradient, with younger age among African American women, is consistent with intersectional vulnerability in the reproductive period [14,15]. In contrast, the markedly higher HCM prevalence among Caucasians suggests combined contributions of genetic architecture, ascertainment, and access to specialized imaging/interpretation required for diagnosis, which may be inequitably distributed [4,9,16-18].

Mechanistic interpretation

Biological embedding of social adversity provides a potential mechanistic framework: chronic stress, neighborhood disadvantage, and structural exclusion may accelerate cardiometabolic injury via inflammation, oxidative stress, and telomere dynamics, potentially yielding earlier disease expression [6-8,15,19]. Health-system factors, including underrepresentation in trials, clinician bias affecting communication and treatment, and geographic maldistribution of specialty care, are likely to further propagate inequities in detection and therapy [4,16-18]. The convergence of earlier age at onset, greater DCM burden, and male predominance in DCM/HCM highlights the need for tailored prevention and clinical pathways attentive to both social risk and sex-specific biology [14,15,20-23].

Public health implications

Potential public health and clinical implications include earlier risk identification among younger African American adults, pregnancy-adjacent screening for PPCM risk, and investments that may reduce diagnostic deserts for cardiomyopathies requiring advanced imaging. Equity-centered strategies, policy interventions addressing structural racism and access, improved trial inclusion, and bias-aware care delivery may serve as essential complements to guideline-directed therapy [1,4,6-9].

Limitations

This study has several important limitations to consider when interpreting the findings.

Study Design

The cross-sectional design precludes causal inference; observed associations cannot establish that demographic factors or social determinants directly cause differences in cardiomyopathy etiology or age at presentation. Longitudinal studies are needed to establish temporal relationships and causality.

Selection Bias

The dataset comprises patients who underwent cardiac transplantation, LVAD placement, or cardiac biopsy, representing individuals with advanced disease who had access to tertiary care centers. This introduces substantial selection bias, as patients who died before reaching transplant centers, lacked insurance or referral access, or received care in nonacademic settings are not represented. The control group similarly reflects donor characteristics that may not be representative of the general population. These selection mechanisms may differentially affect racial and socioeconomic groups, potentially underestimating true disparities in access and outcomes.

Sample size constraints

Subgroup analyses were limited by small cell sizes, particularly for PPCM (n = 6 overall, n = 2 African American) and African American HCM cases (n = 1), precluding robust statistical inference for these comparisons. Confidence intervals for these estimates are wide, and findings should be considered preliminary pending replication in larger cohorts.

Limited Sociodemographic Granularity

The dataset lacked detailed SDoH variables, including education, income, insurance status, neighborhood deprivation indices, and healthcare access measures. Race categories were limited to African Americans and Caucasians, excluding Hispanic, Asian, and other populations. The reliance on broad racial categories and the absence of socioeconomic data prevented multivariable modeling to disentangle the independent contributions of race, socioeconomic status, and structural factors to observed disparities. Additionally, race and sex were based on available metadata without information on data collection methods or participant self-identification.

Generalizability

The findings may not generalize to community-based populations with less severe disease, other racial/ethnic groups, or international settings with different healthcare systems and social structures. The dataset's geographic and temporal scope is not fully detailed in the public repository, limiting assessment of secular trends or regional variation.

Unmeasured Confounders

Important clinical factors such as comorbidity burden, medication adherence, time from symptom onset to diagnosis, and quality of care received before transplant referral were not available and may confound observed associations.

Despite these limitations, the effect sizes were large and statistically robust for the primary comparisons, and findings align with the broader cardiovascular disparities literature. The etiology-specific insights extend prior work by demonstrating that demographic patterns vary meaningfully across cardiomyopathy subtypes, emphasizing the need for tailored equity interventions.

## Conclusions

Demographic patterns in cardiomyopathy etiologies are associated with SDoH. African Americans are more likely to experience substantially greater and earlier DCM, whereas HCM is far more common among Caucasians; male sex is overrepresented in both DCM and HCM. These intersectional patterns indicate that prevention, diagnostic access, and treatment may need to be redesigned to meet the needs of socially vulnerable groups earlier in the life course.

Achieving cardiovascular health equity will require integrating upstream structural interventions with downstream clinical excellence. Equity-centered screening, expanded access to specialized diagnostics, and inclusive research participation are immediate levers that may help reduce the disproportionate burden borne by marginalized communities. Future research should examine longitudinal trajectories, including comprehensive SDoH measures, and test interventions designed to address the structural and systemic factors underlying these disparities.
